# Normalization of obstructive cardiomyopathy and improvement of hepatopathy on ketogenic diet in patient with glycogen storage disease (GSD) type IIIa

**DOI:** 10.1016/j.ymgmr.2020.100628

**Published:** 2020-07-16

**Authors:** Tatiana Marusic, Mojca Zerjav Tansek, Andreja Sirca Campa, Ajda Mezek, Pavel Berden, Tadej Battelino, Urh Groselj

**Affiliations:** a University Children's Hospital, University Medical Center Ljubljana, Bohoričeva ulica 20, Ljubljana, Slovenia; b Clinical Institute of Radiology, University Medical Center Ljubljana, Zaloška cesta 7, 1000 Ljubljana, Slovenia; c Faculty of Medicine, University of Ljubljana, Vrazov trg 2, 1000 Ljubljana, Slovenia

**Keywords:** Glycogen storage disease, GSD type IIIa, Cardiomyopathy, Ketogenic diet, Case report, GSD, Glycogen Storage Disease, KD, Ketogenic diet, KB, Ketone bodies, LVMI, Left Ventricular Mass Index, MAD, Modified Atkins Diet, MRI, Magnetic Resonance Imaging, CK, Creatine kinase, proBNP, pro-Brain-Type Natriuretic Peptide

## Abstract

Now 15-year-old girl with glycogen storage disease (GSD) type IIIa (OMIM 232400) developed severe left ventricular obstructive hypertrophy and hepatomegaly while treated with frequent cornstarch meals. Subsequently, she was introduced the ketogenic diet; continuous ketosis has been maintained for over the last 4 years. After the introduction of ketogenic diet, a normalization of the cardiomyopathy and improvement of hepatopathy was achieved, with enhanced overall quality of life.

## 1 Introduction

Glycogen storage disease (GSD) type III (OMIM 232400) is an autosomal recessive inherited error of metabolism caused by the deficiency of glycogen debranching enzyme amylo-1,6-glucosidase, encoded by *AGL* gene, resulting in an incomplete glycogen degradation and its accumulation. The estimated GSD type III incidence is 1/100,000 live births. In addition to hepatomegaly, which is present in GSD type IIIb, also variable skeletal myopathy and cardiomyopathy are characteristic for GSD type IIIa, possibly leading to a cardiac failure [1]. Traditionally, the dietary treatment of the disease based on adding cornstarch and/or continuous night-time feeds with oligosaccharides (1.6 g per kg body weight (g/kg) every 4 h for an infant or young child or 1.7–2.5 g/kg every 6 h for an older child) to prevent hypoglycemia. However, the diet rich in carbohydrates further contributes to glycogen accumulation in tissues and to hyperinsulinism [2,3]. To date, there are no universally accepted recommendations for the use of ketogenic diet (KD) in GSD type IIIa.

We aimed to assess the influence of KD on the severe obstructive cardiomyopathy, hepatopathy and other clinical characteristics in a patient with GSD type IIIa. We made a decision to introduce KD in our patient due to her rapidly worsening condition, basing on a few promising reports that were then available [[4], [5], [6]].

## 2 Material and methods

This is a case report of a now 15-year old girl with GSD type IIIa (with previously confirmed *AGL* gene homozygous mutation c.3980G>A (p.W1327X) resulting in an almost undetectable amylo-1,6-glucosidase activity), who was diagnosed at 1 year of age. At that time she was introduced a high carbohydrates diet (frequent diurnal and nocturnal cornstarch meals); carbohydrates (9 g/kg per day (g/kg/d)) contributed 53% daily calories, proteins (4 g/kg/d) contributed 23% and fats (1.8 g/kg/d) contributed another 23%. Progressively she developed left ventricular obstructive hypertrophy, hepatomegaly and skeletal myopathy with highly elevated liver and muscle enzymes. She also presented recurrent hypoglycemic events despite treatment with frequent diurnal and nocturnal meals with cornstarch supplements. Due to progressive obstructive cardiomyopathy, she was introduced KD at the age of 11. The diet consisted of ketogenic ratios of 2.5:1; fats (5.2 g/kg/d) contributed 87% daily calories, proteins (1.6 g/kg/d) contributed 11% and carbohydrates (0.3 g/kg/d) contributed 2%. Continuous ketosis was maintained for over 4 years. A very good clinical support by attending physicians and experienced clinical dietitians was provided (e.g. helping with practical dilemmas via e-mail/phone soon after they arise), also to avoid discontinuation of ketosis. The blood sampling at regular outpatient visits was performed in a fasting state, as recommended [3]. For home monitoring, she daily measured the ketones in urine using a semiquantitative test.

## 3 Results

**Fig. 1:**
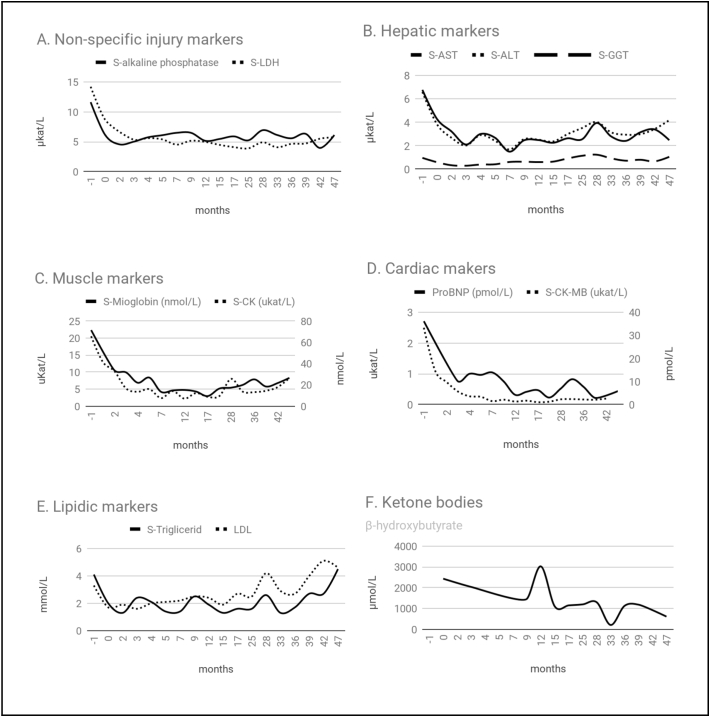
The impact of ketogenic diet (KD) on selected cardiac and hepatic-metabolic biochemical biomarkers (setting 0 months as the KD introduction).

Within a few months after KD introduction, laboratory parameters improved significantly. Cardiac enzymes and congestive heart failure markers dropped after KD introduction. (Fig. 1A, C and D). Furthermore, hepatic injury markers decreased more than twice (Fig. 1B). Regarding lipids, the levels decreased at the beginning, with a later slightly increased, related to a high fat diet (Fig. 1E).

Exertion dyspnea disappeared, while capacity for oxygen consumption almost doubled at control examination. Abdominal ultrasound showed a significant improvement of hepatomegaly; the standard liver measures sharply decreased in 6 months after the incorporation of KD (sternal line 129 to 110 mm; medioclavicular line 162 to 137 mm; anterior axillary line 167 to 146 mm) and sustained in normal range after 4 years.

Cardiac MRI was repeated after 16 and after 40 months, showing a normalization of left ventricular parameters, with a decreased of total left ventricular mass index (LVMI) (from 58 g/m^2^ to 37 g/m^2^) and thickness of left ventricular walls (lateral wall from 10 to 5 cm, septal wall from 9 to 5 cm and inferoseptal wall from 10 to 7 cm), without residual outflow obstruction. Finally, no hypoglycemic events were recorded while on KD.

## 4 Discussion

While on KD in our patient, we observed normalization of severe progressive obstructive cardiomyopathy, similarly as reported in a few previous shorter observations. Dagli et al. report on a male child with GSD type IIIa, treated with a high carbohydrate diet, developing severe cardiomyopathy with LVMI of 209 g/m^2^. At the age of 22, with a diet consisting of a protein intake representing 30% of total energy, he achieved a complete improvement in the cardiomyopathy (LVMI decreasing from 160 g/m^2^ to 78 g/m^2^) [4]. Mayorandan et al. reports on two boys with GSD type IIIa aged 9 and 11 years, who started treatment with hyperproteic and cornstarch meals. One of the patients, however, in the first years of life quickly developed severe left ventricular hypertrophy; while the second patient presented minor concentric hypertrophic cardiomyopathy with a prolonged QTc-time. After that they started a Modified Atkins diet (MAD), a variant of KD with similar efficacy. One year later both had an improvement of CK levels, congestive heart failure markers and symptoms disappeared. Patient 1 presented a decrease of 80% in CK levels; and in 3 years the proBNP levels dropped more than 90%. An important reduction of the thickness of interventricular septum and left ventricle posterior wall and an improvement of the outflow obstruction was shown in the ultrasound. Patient 2 had irregular adherence, but in the periods he committed to the diet he presented a reduction of CK levels from 88.2 to 51 ukat/L in one month [5]. In addition, Brambilla et al. reports on two siblings (five and seven years old) with GSD type IIIa treated with a MAD over a period of 32 and 26 months, respectively. In both patients, CK levels in blood decreased, cardiomyopathy improved and associated symptoms disappeared [6]. Furthermore, a recent cohort study by Rossi et al. reveals a decrease of CK levels in 89% of patients with GSD type IIIa treated with high fat diet [7]. Francini-Pesenti et al. report on a 34 years-old patient with GSD type IIIa with hypertrophic cardiomyopathy who was also treated with a MAD. After 12 months of treatment, ejection fraction raised from 30% to 45% and physical activity increased more than twice [8]. Our patient maintained an ejection fraction between 85% and 69%.

Regarding hepatopathy, Rossi et al. also report a decrease of ALT and AST concentrations in adults in 100% and 83% patients, respectively. In children, ALT and AST decrease in only 11% and 22% patients, respectively [7]. An improvement of ALT and AST levels is reported by Brambilla (AST of 21% and 72%; ALT of 6 and 38%) and Francini-Pesenti (AST of 29% and ALT of 24%), but they do not report any change in liver sizes [6,8]. However, in our patient the liver functions improved (from 6.78 to 2.49 ukat/L and ALT from 6.6 to 2.2 ukat/L) and organ-measures have substantially decreased (approximately 2 cm). The liver biopsy was not performed in our case which would allow the assessment of the liver fibrosis. Halaby et al. recommend active hepatic disease surveillance, as liver complications could be silent and underestimated [9]. They suggest the measure of urinary glucose tetrasaccharide (Glc4) as an hepatic glycogen storage marker. Although further studies are needed to verify its specificity, it could be a promising noninvasive tool for monitoring liver disease progression.

Development of the cardiomyopathy in GSD type IIIa may be caused by the high carbohydrate diet, according to reports of the literature about the association between carbohydrate overtreatment and an elevated risk for cardiac involvement and/or cardiomyopathy [1]. Rossi et al. reports that an early switch to a high fat diet decreases cardiac glycogen storage, probably caused by lower carbohydrate intake and fat properties [7]. Furthermore, it is hypothesized that elevated levels of insulin, generated by a high carbohydrate diet, activate glycogen synthase and promote glycogen storage in myocytes and lipolysis inhibition [5]. On the other hand, ketone bodies (KB) might revert glycogen deposits. De Cabo et al. recently described the benefits of KB in a study about long periods of fasting. Besides the energetic role during these periods, the KB are powerful signaling molecules with significant effects on cell and organ functions. They regulate the expression and activity of many proteins and molecules involved in systemic metabolism, impacting aging and health [10]. Furthermore, ketosis is known to activate mitochondrial succinate dehydrogenase in the heart thereby improving the energetic balance [11]. The KB have also been widely studied for treatment of epilepsy and several studies in children confirmed its efficacy [12].

Some concerns were previously expressed regarding the low expected adherence to KD for these patients [5,6]. The previously mentioned cases reports were treated with MAD, which doesn't need to be mathematically pre-calculated. The classical ketogenic diet must be individually calculated and medically monitored. However, our patient has had a very good overall compliance to the KD. In addition, the only side effects observed were mildly-to-moderately elevated lipid levels, as was also observed previously in other patients [12] (Fig. 1E). Halaby et al. highlighted the relevance of LDL/HDL ratio and BMI control for monitoring of cardiovascular disease progression [9].

During the 4-year follow up, the patient and her family reported much better quality of life while on KD; reasons stated were: no need for burdensome overnight dietary regimen; no hypoglycemic events recorded; much improved physical fitness and self-image after losing weight; and most importantly, to worrying less about the worsening cardiomyopathy. This further reinforces our opinion about KD feasibility and efficacy.

## 5 Conclusions

Cardiomyopathy is a potentially life-threatening complication in treated GSD IIIa patients that could be reversed on the KD, which has also potential to significantly improve other clinical characteristics and overall quality of life, with a good safety profile. KD was shown to be feasible for the patient and her family, which resulted in a good compliance to the diet. It is worth mentioning that our reported study has the longest follow-up to date. Further prospective studies are needed; however, the data yet available might already suffice to be encompassed into the recommendations on GSD type IIIa management strategies.

## Funding

This research did not receive any specific grant from funding agencies in the public, commercial, or not-for-profit sectors. Study was partly founded by the 10.13039/501100004329Slovenian Research Agency project V3--1505 and program P3--0343.

## References

[1] Sentner C.P., Hoogeveen I.J., Weinstein D. (2016). Glycogen storage disease type III: diagnosis, genotype, management, clinical course and outcome. J. Inherit. Metab. Dis..

[2] Kishnani P.S., Austin S.L., Arn P., Bali D.S., Boney A., Case L.E. (2010). Glycogen storage disease type III diagnosis and management guidelines. Genitourin. Med..

[3] Dagli A., Sentner C., Weinstein D.A. (2010). Glycogen storage disease type III. GeneReviews.

[4] Dagli A.I., Zori R.T., McCune H., Ivsic T., Maisenbacher M.K., Weinstein D.A. (2009). Reversal of glycogen storage disease type IIIa –related cardiomyopathy with modification of diet. J. Inherit. Metab. Dis..

[5] Mayorandan S., Meyer U., Hartmann H. (2014). Glycogen storage disease type III: modified Atkins diet improves myopathy. Orphanet J. Rare Dis..

[6] Brambilla A., Mannarino S., Pretese R. (2014). Improvement of cardiomyopathy after high-fat diet in two siblings with glycogen storage disease type III. JIMD Rep..

[7] Rossi A., Hoogeveen I.J., Bastek V.B., de Boer F., Montanari C., Meyer U. (2020). Dietary lipids in glycogen storage disease type III: a systematic literature study, case studies and future recommendations. JIMD.

[8] Francini-Pesenti F., Tresso S., Vitturi N. (2019). Modified Atkins ketogenic diet improves heart and skeletal muscle function in glycogen storage disease type III. Acta Myol..

[9] Halaby C.A., Young S.P., Austin S., Stefanescu E., Bali D., Clinton L.K. (2019). Liver fibrosis during clinical ascertainment of glycogen storage disease type III: a need for improved and systematic monitoring. Genet. Med..

[10] De Cabo R., Mattson M. (2019). Effects of intermittent fasting on health, aging, and disease. N. Engl. J. Med..

[11] Balietti M., Fattoretti P., Giorgetti B., Casoli T., Di Stefano G., Solazzi M., Platano D., Aicardi G., Bertoni-Freddari C. (2009). A ketogenic diet increases succinic dehydrogenase activity in aging cardiomyocytes. Ann. N. Y. Acad. Sci..

[12] Kossoff E., Rowley H., Sinha S., Vining E.P. (2008). A prospective study of the modified Atkins diet for intractable epilepsy in adults. Epilepsia.

